# Chapter 14: Cancer Genome Analysis

**DOI:** 10.1371/journal.pcbi.1002824

**Published:** 2012-12-27

**Authors:** Miguel Vazquez, Victor de la Torre, Alfonso Valencia

**Affiliations:** Structural Biology and BioComputing Programme, Spanish National Cancer Research Centre (CNIO), Madrid, Spain; Whitehead Institute, United States of America; University of Maryland, Baltimore County, United States of America

## Abstract

Although there is great promise in the benefits to be obtained by analyzing cancer genomes, numerous challenges hinder different stages of the process, from the problem of sample preparation and the validation of the experimental techniques, to the interpretation of the results. This chapter specifically focuses on the technical issues associated with the bioinformatics analysis of cancer genome data. The main issues addressed are the use of database and software resources, the use of analysis workflows and the presentation of clinically relevant action items. We attempt to aid new developers in the field by describing the different stages of analysis and discussing current approaches, as well as by providing practical advice on how to access and use resources, and how to implement recommendations. Real cases from cancer genome projects are used as examples.

**What to Learn in This Chapter**

This chapter presents an overview of how cancer genomes can be analyzed, discussing some of the challenges involved and providing practical advice on how to address them. As the primary analysis of experimental data is described elsewhere (sequencing, alignment and variant calling), we will focus on the secondary analysis of the data, *i.e.*, the selection of candidate driver genes, functional interpretation and the presentation of the results. Emphasis is placed on how to build applications that meet the needs of researchers, academics and clinicians. The general features of such applications are laid out, along with advice on their design and implementation. This document should serve as a starter guide for bioinformaticians interested in the analysis of cancer genomes, although we also hope that more experienced bioinformaticians will find interesting solutions to some key technical issues.

This article is part of the “Translational Bioinformatics” collection for *PLOS Computational Biology*.

## 1. Introduction

Cancer is commonly defined as a “disease of the genes”, a definition that emphasizes the importance of cataloguing and analyzing tumor-associated mutations. The recent advances in sequencing technology have underpinned the progress in several large-scale projects to systematically compile genomic information related to cancer. For example, the Cancer Genome Atlas (http://cancergenome.nih.gov/) and the projects overseen by the International Cancer Genome Consortium [1] (http://icgc.org/) have focused on identifying links between cancer and genomic variation. Unsurprisingly, the analysis of genomic mutations associated with cancer is also making its way into clinical applications [2]–[4].

Cancer may be favored by genetic predisposition, although it is thought to be primarily caused by mutations in specific tissues that accumulate over time. Genetic predisposition is represented by germline variants and indeed, many common germline variants have been associated with specific diseases, as well as with altered drug susceptibility and/or toxicity. The association of germline variants with clinical features and disease is mainly achieved through Genome Wide Association Studies (GWAS). GWAS use large cohorts of cases to analyze the relationship between the disease and thousands or millions of mutations across the entire genome, and they are the subject of a separate chapter in this issue.

The study of cancer genomes differs significantly from GWAS, as during the lifetime of the organism variants only accumulate in the tumor or the affected tissues, and they are not transmitted from generation to generation. These are known as somatic mutations. Mutations accumulate as the tumors progress through processes that are not completely understood and that depend on the evolution of the different cell types in the tumor, *i.e.*, clonal versus parallel evolution [5]. Regardless of which model is more relevant, the tumor genome includes mutations that facilitate tumorigenesis or are that essential for the generation of the tumor (known as tumor ‘drivers’), and others that have accumulated during the growth of the tumor (known as ‘passengers’) [6]. Distinguishing ‘driver’ from ‘passenger’ mutations is crucial for the interpretation of cancer genomes [5].

Depending on the type of data and the aim of the analysis, cancer genome analysis may focus on the cancer type or on the patient. The first approach consists of examining a cohort of patients suffering from a particular type of cancer, and is used to identify biomarkers, characterize cancer subtypes with clinical or therapeutic implications, or to simply advance our understanding of the tumorigenic process. The second approach involves examining the genome of a particular cancer patient in the search for specific alterations that may be susceptible to tailored therapy. Although both approaches draw on common experimental and bioinformatics techniques, they analyze different types of information, have different goals and they require the presentation of the results in distinct ways.

The development of Next Generation Sequencing (NGS) has not only helped identify genetic variants but also, it represents an important aid in the study of epigenetics (DNAseq and ChipSeq of histone methylation marks), transcriptional regulation and splicing (RNAseq). The combined power of such genomic data provides a more complete definition of ‘cancer genomes’.

To aid developers new to the field of cancer genomics, this chapter will discuss the particularities of cancer genome analysis, as well as the main scientific and technical challenges, and potential solutions.

## 2. Overview of Cancer Genome Analysis

**Figure 1:**
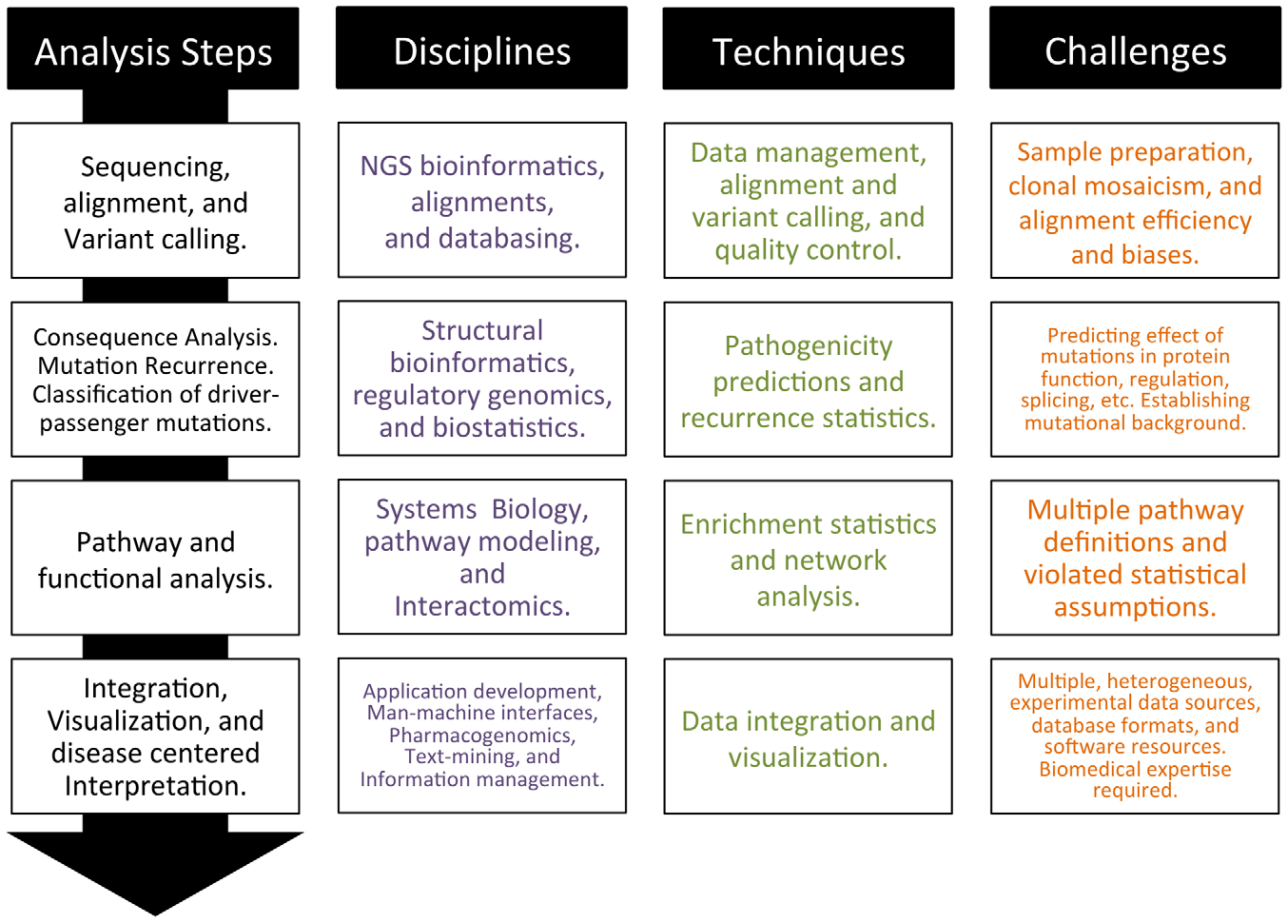
Idealized cancer analysis pipeline. The column on the left shows a list of sequential steps. The columns on the right show the bioinformatics and molecular biology disciplines involved at each step, the types of techniques employed and some of the current challenges faced.

The sequence of the steps in an idealized cancer genome analysis pipeline are presented in Figure 1. For each step listed, the biological disciplines involved, the bioinformatics techniques used and some of the most salient challenges that arise are listed.

### 2.1. Sequencing, Alignment and Variant Calling

After samples are sequenced, sequencing reads are aligned to a reference genome and all differences are identified through a process known as *variant calling*. The output of the variant calling is a list of genomic variations that is organized according to their genomic location (chromosome and position) and the variant allele. They may be accompanied by scores measuring the sequencing quality over that region or the prevalence of the variant allele in the samples. The workflow employed for this type of analysis is commonly known as a primary analysis (For more information on sequencing, alignment and variant calling, please refer to [7], [8]).

This chapter describes the subsequent steps in the analysis of the variants detected at the genome level. This process is relatively well established and is the main subject of this chapter.

### 2.2. Consequence, Recurrence Analysis and Candidate Drivers

The list of somatic variants obtained from the primary analysis of DNA sequences is carefully examined to identify mutations that may alter the function of protein products. DNA mutations are translated into mutations in RNA transcripts, and from RNA into proteins, potentially altering their amino acid sequence. The impact of these amino acid alterations on protein function can range from largely irrelevant (if they do not affect any region of the protein involved in catalysis or binding, or if they do not significantly alter the structure and stability of the protein) to highly deleterious (for example if the amino acid changes result in the formation of a truncated protein lacking important functional regions). The severity of these alterations can be assessed using specialized software tools known as *protein mutation pathogenicity predictors*.

Mutations are also examined to identify recurrence, which may point to key genes and mutational hotspots. The predicted consequences of the mutations and their recurrence are used to select potential driver mutations that may be directly involved in the tumorigenic process.

Note that not all mutations that have deleterious consequences for protein function are necessarily involved in cancer as the proteins affected may not play any fundamental role in tumorigenesis.

### 2.3. Pathways and Functional Analysis

Genes that are recurrently mutated in cancer tend to be easily identifiable, and obvious examples include TP53 and KRAS that are mutated in many cancer types. More often mutations are more widely distributed and the probability of finding the same gene mutated in several cases is low, making it more difficult to identify common functional features associated with a given cancer.

Pathway analysis offers a means to overcome this challenge by associating mutated genes with known signaling pathways, regulatory networks, clusters in protein interaction networks, protein complexes or general functional classes, such as those defined in the Gene Ontology database. A number of statistical methods have been developed to determine the significance of the associations between mutated genes and these functional classes. Pathways analysis has now become a fundamental component of cancer genome analysis and it is described in almost all cancer genome publications. In this sense, cancer is not only a ‘disease of the genes’ but also a ‘disease of the pathways’.

### 2.4. Integration, Visualization and Interpretation

Information on the mutational status of genes can be better understood if it is integrated with information about gene expression and related to alterations in: the copy number of each gene (CNVs), a very common phenomenon in cancer; mutations in promoters and enhancers; variations in the affinity of transcription factors and DNA binding proteins; or dysregulation of epigenetic control.

The importance of the relationships between different genome data sources is illustrated by the case of chronic lymphocytic leukemia (CLL). The consequences of mutations in the SF3B1 splicing factor, detected by exon sequencing [9], were investigated in studies of DNA methylation [10] and RNA sequencing in the same patients (Ferreira et al. submitted). At the technical level, the analysis of heterogeneous genomic data adds further complications to analysis workflows, as the underlying biological bases are often not fully understood. Consequently, relatively few published studies have effectively combined more than a few combinations of such data [11]–[13]. These studies are usually supported by visualization tools to analyze the results within specialist applications tailored to fit the specific set of data generated.

Finally, in a personalized medicine application, the results must be related to information of clinical relevance, such as potentially related drugs and therapies.

### 2.5. Current Challenges

In general terms, three key challenges exist when analyzing cancer genomes: (1) the heterogeneity of the data to be analyzed, which ranges from genomic mutations in coding regions to alterations in gene expression or epigenetic marks; (2) the range of databases and software resources required to analyse and interpret the results; and (3) the comprehensive expertise required to understand the implications of such varied experimental data.

## 3. Critical Bioinformatics Tasks in Cancer Genome Analysis

**Figure 2:**
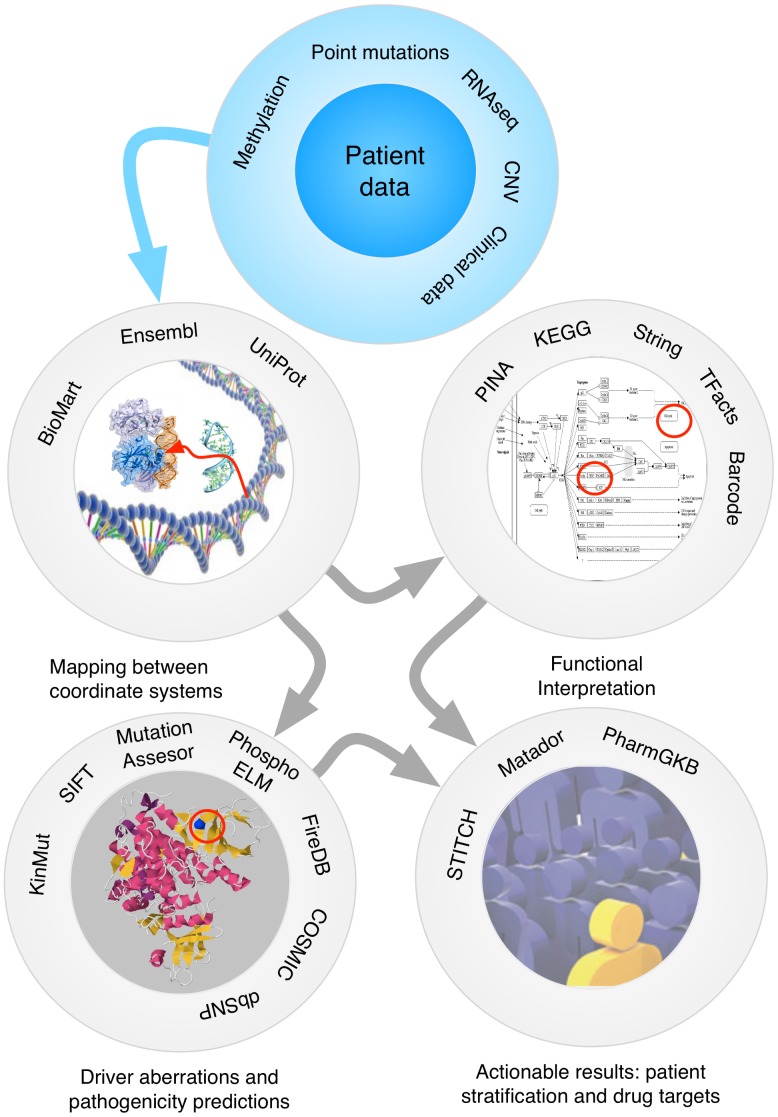
Main tasks in an analysis pipeline. Starting with the patient information derived from NGS experiments, the variants are mapped between genes and proteins, evaluated for pathogenicity, considered systemically through functional analysis, and the resulting conclusions translated into actionable results.

An overview of the four main tasks that should be performed when analyzing the cancer genome is shown in Figure 2, along with the associated requirements. In the first instance, the mutations initially detected at the DNA level must be trimmed to include only somatic variations, removing the germline SNPs detected in healthy tissue of the same individuals or in the general population. The description of the different stages of analysis that we present begins with this list of somatic variants and their associated genomic locations.

### 3.1. Mapping between Coordinate Systems

Translating mutational information derived from genomic coordinates to other data types is an obvious first step. Although this may seem trivial, its importance should not be underestimated given that alterations in single nucleotides can have significant consequences.

The position of DNA mutations in transcripts and protein products must be obtained by translating their coordinates across various systems. For example, point mutations in coding regions can be mapped to different transcripts by finding the exon affected, the offset of the mutation inside that exon and the position of the exon inside the transcript. By removing the 5′ UTR region of the transcript sequence and dividing the rest into triplets, the affected codon can be identified, as well as the possible amino acid replacement. Ensembl BioMart provides all the information necessary to perform this type of mapping, while a number of other systems also provide this functionality (see Table 1).

**Table 1:** Selection of the software packages used in cancer genome analysis.

Software	Functionality	Availability
VEP	Mutation mapping	Local installation or web site
ANNOVAR	Mutation mapping	Local installation
VARIANT	Mutation mapping	Local installation, web site, and web service
Mutation Assessor, SIFT	For protein variants	Web site and web service
Condel	Consensus prediction	Web site and web service
wKinMut	Kinase specific	Web site and web service
Genecodis	Annotation enrichment for gene lists	Web site and web service
FatiGO, David	Annotation enrichment for gene lists	Web site
Cytoscape	Network visualization and analysis	Local installation. Can be embedded in browser applications
R	Statistics and plotting	Local installation
Taverna	Workflow enactment	Local installation
Galaxy	Workflow enactment	Browser application

One important technical consideration when mapping genomic variants is the version of the genome build. It is essential to use the correct build and many mapping tools support different versions of the genome build. Moreover, the data in Ensembl is thoroughly versioned, so that the BioMart interface can be used to gather all genomic information consistently for any particular build. Thus, entities (mutations, genes, transcripts or proteins) can be linked back to the appropriate version using the Ensembl web site archives.

### 3.2. Driver Mutations and Pathogenicity Prediction

In addition to false variants introduced by technical errors, some variants present in the samples may not contribute to cancer development. The terms ‘driver’ and ‘passenger’ were first used in 1964 in the context of viral infections that drive cancer [6]. However, they are now used to distinguish mutations that drive cancer onset and progression from those that play little or no role in such processes but that are propagated by their co-existence with driver mutations. The problem of distinguishing driver from passenger mutations remains unsolved as yet. Experimental assays of activity are one means of testing the tumorigenic potential of mutations [14], although such assays are difficult to perform to scale. Consequently, a number of complementary *in-silico* methods have been developed to identify driver mutations. Statistical approaches seek to identify traces of mutation selection during tumor formation by looking at the prevalence of mutations in particular genes in sample cohorts, or the ratios of synonymous versus non-synonymous mutations in particular candidate genes. However, such statistical approaches require large sample cohorts to achieve sufficient power. Alternatively, *in-silico* predictions of pathogenicity can be used to restrict the list of potential driver mutations to those that are likely to alter protein function [15].

Several tools that implement different versions of these general concepts can be used to perform pathogenicity predictions for point mutations in coding regions (see Table 1). Prediction is far more complicated for genomic aberrations and mutations that affect non-coding regions of DNA, an area of basic research that is still in its early stages. However, the large collections of genomic information gathered by the ENCODE project [16] will doubtless play a key role in this research.

Despite their limited scope, mutations in coding regions are the most useful for cancer genome analysis. This is initially because it is still cheaper to sequence exomes than full genomes and also, because they are closer to actionable medical items, given that most drugs target proteins. Indeed, most clinical success stories based on cancer genome analysis have involved the analysis of point mutations in proteins [3].

In particular, we have focused on the need to analyze the consequences of mutations in alternative isoforms of each gene, in addition to those in the main isoforms. Despite the potential implications of alternative splicing, this problem remains largely overlooked by current applications. A common solution is to assign the genomic mutations to just one of the several potential isoforms, without considering their possible incidence of other splice isoforms, and in most cases without knowing which isoform is actually produced in that particular tissue. The availability of RNAseq data should solve this problem by demonstrating which isoforms are specifically expressed in the cell type of interest, in which case, additional software will be necessary to analyze the data generated by the new experiments.

### 3.3 Functional Interpretation

Some genes harbor a large number of mutations in cancer genomes, such as TP53 and KRAS, whose importance and relevance as cancer drivers have been well established. Frequently however, genomic data reveals the presence of mutated genes that are far less prevalent, and the significance of these genes must be considered in the context of the functional units they are part of. For example, SF3B1 was mutated in only 10 out of 105 samples of chronic lymphocytic leukemia (CLL) in the study conducted by the ICGC consortium [9], and in 14 out of 96 in the study performed in the Broad Institute [17]. While these numbers are statistically significant, many other components of the RNA splicing and transport machinery are also mutated in CLL. Even if these mutations occur at lower frequencies they further emphasize the importance of this gene [18].

Functional interpretation aims to identify large biological units that correlate better with the phenotype than individual mutated genes, and as such, it can produce a more general interpretation of the acquired genomic information. The involvement of genes in specific biological, metabolic and signaling pathways is the type of functional annotation most commonly considered and thus, functional analysis is often termed ‘pathway analysis’. However, functional annotations may also include other types of biological associations such as cellular location, protein domain composition, and classes of cellular or biochemical terms, such as GO terms (Table 2 lists some useful databases along with the relevant functional annotations).

**Table 2:** Selection of databases commonly used in our workflows.

Database	Entities	Properties
Ensembl	Genes, proteins, transcripts, regulatory regions, variants	Genomic positions, relationships between them, identifiers in different formats, GO terms, PFAM domains
Entrez	Genes, articles	Articles for genes, abstracts of articles, links to full text
UniProt	Proteins	PDBs, known variants
KEGG, Reactome, Biocarta, Gene Ontology	Genes	Pathways, processes, function, cell location
TFacts	Genes	Transcription regulation
Barcode	Genes	Expression by tissue
PINA, HPRD, STRING	Proteins	Interactions
PharmaGKB	Drugs, proteins, variants	Drug targets, pharmacogenetics
STITCH, Matador	Drugs, proteins	Drug targets
Drug clinical trials	Investigational drugs	Diseases or conditions in they are being tested
GEO, ArrayExpress	Genes (microarray probes)	Expression values
ICGC, TCGA	Cancer Genomes	Point mutations, methylation, CNV, structural variants
dbSNP, 1000 genomes	Germline variations	Association with diseases or conditions
COSMIC	Somatic variations	Association with cancer types

Over the last decade, multiple statistical approaches have been developed to identify functional annotations (also known as ‘labels’) that are significantly associated with lists of entities, collectively known as ‘enrichment analysis’. Indeed, the current systems for functional interpretation have been derived from the systems previously developed to analyze expression arrays, and they have been adapted to analyze lists of cancer-related genes. As this step is critical to perform functional interpretations, special care must be taken when selecting methods to be incorporated into the analysis pipeline. Cases in which the characteristics of the data challenge the assumptions of the methods are particularly delicate. For instance, a hyper-geometric test might be appropriate to analyze gene lists that are differentially expressed in gene expression arrays. However, when dealing with lists of mutated genes this approach does not account for factors such as the number of mutations per gene, the size of the genes, or the presence of genes in overlapping genomic clusters (where one mutation may simultaneously affect several genes). As none of these issues are accommodated by the standard approaches used for gene expression analysis, new developments are clearly required for cancer genome analysis.

To alleviate the rigidity introduced by the binary nature of set-based approaches, whereby genes are either on the list or they are not, some enrichment analysis approaches study the over-representation of annotations/labels using rank-based statistics. A common choice for rank-based approaches is to use some variation of the Kolmogorov-Smirnov non-parametric statistic, as employed in gene set enrichment analysis (GSEA) [19]. Another benefit of rank approaches is that the scores used can be designed to account for some of the features that are not well handled by set-based approaches. Accordingly, considerations of background mutation rates based on gene length, sequencing quality or heterogeneity in the initial tumor samples can be incorporated into the scoring scheme. However, rank statistics are still unable to handle other issues, such as mutations affecting clusters of genes that are functionally related (*e.g.*, proto-cadherins), which still challenge the assumption of independence made by most statistical approaches. Note that from a bioinformatics perspective, sets of entities are often conceptually simpler to work with than ranked lists when crossing information derived from different sources. Moreover, from an application perspective, information summarized in terms of sets of entities is often more actionable than ranks or scores.

A different type of analysis considers the relationships between entities based on their connections in protein interaction networks. This approach has been used to measure the proximity of groups of cancer-related genes and other groups of genes or functions, by labeling nodes with specific characteristics (such as roles in biological pathways or functional classes) [20].

Functional interpretation can therefore be facilitated by the use of a wide array of alternative analyses. Different approaches can potentially uncover hidden functional implications in genomic data, although the integration of these results remains a key challenge.

### 3.4. Applicable Results: Diagnosis, Patient Stratification and Drug Therapies

For clinical applications, the results of cancer genome analysis need to be translated into practical advice for clinicians, providing potential drug therapies, better tumor classification or early diagnostic markers. While bioinformatics systems can support these decisions, it will be up to expert users to present these findings in the context of the relevant medical and clinical information available at any given time. In the case of our institution's (CNIO) personalized cancer medicine approach, we use mouse xenografts (also known as ‘avatar’ models) to test the effects of drugs on tumors prior to considering their potential to treat patients [4]. In turn, the results of these xenograft studies are used as a feedback into the system for future analyses.

Drug-related information and the tools with which to analyze it is essential for the analysis of personalized data (some of the key databases linking known gene variants to diseases and drugs are listed in Table 2). Accessing this information and integrating chemical informatics methodologies into bioinformatics systems presents new challenges for bioinformaticians and system developers.

## 4. Resources for Genome Analysis in Cancer

### 4.1. Databases

Although complex, the data required for genome analysis can usually be represented in a tabular format. Tab separated values (TSV) files are the *de facto* standard when sharing database resources. For a developer, these files have several practical advantages over other standard formats popular in computer science (namely XML): they are easier to read, write and parse with scripts; they are relatively succinct; the format is straight-forward and the contents can be inferred from the first line of the file, which typically holds the names of the columns.

Some databases describe entities and their properties, such as: proteins and the drugs that target them; germline variations and the diseases with which they are associated; or genes along with the factors that regulate their transcription. Other databases are repositories of experimental data, such as the Gene Expression Omnibus and ArrayExpress, which contain data from microarray experiments on a wide range of samples and under a variety of experimental conditions. For cancer genome studies, cancer-specific repositories will soon be the main reference, such as those developed by the ICGC and TCGA projects. Indeed, these repositories contain complete genotypes that offer a perfect opportunity to test new approaches with real data.

Bioinformaticians know that crossing information from different sources is not a trivial task, as different resources use a variety of identifiers. Even very similar entities can have different identifiers in two different databases (*e.g.*, genes in Entrez and Ensembl). Some resources borrow identifiers for their own data, along with HGNC gene symbols, while databases such as KEGG have their own identifiers for genes, and offer equivalence tables that map them to gene symbols or other common formats.

In addition to entities being referenced by different identifier formats, in distinct resources they may also adhere to slightly different definitions (*e.g.*, regarding what constitutes a gene). Furthermore, as mentioned above the differences between genome builds can substantially affect the mapping between coordinate systems, and they can also give rise to differences between entities.

In general, translating identifiers can be cumbersome and incompatibilities may exist between resources. For example, MutationAssessor, which predicts the pathogenicity of protein mutations [15], uses UniProt identifiers. Analysis systems using Ensembl data for coordinate mappings, such as our own, render mutations using Ensembl Protein IDs, and in some cases there are problems in translating identifiers, and even in assigning mutations to the wrong isoforms. To prevent these potential errors, MutationAssessor double checks that the original amino acid matches the sequence it is using and refuses to make a prediction otherwise. Although avoiding incorrect predictions is a valid strategy, in practice it substantially reduces the number of predictions that can be made.

Identifier translation is a very common task in Bioinformatics in general, and in cancer genome analysis in particular. In practice, we use the Ensembl BioMart web service to download identifier equivalence tables (in TSV format), which map different identifier formats between and across genes, proteins, array probes, *etc.* We build fast indexes over these equivalence tables and make them ubiquitously accessible to all our functionalities through simple API calls, web services, or command line statements. While potentially encumbered by semantic incompatibilities between entity definitions in multiple resources, a thoroughly versioned translation equivalence system is an invaluable asset for database integration.

### 4.2. Software Resources

In cancer analysis pipelines, several tasks must be performed that require supporting software. These range from simple database searches to cross-check lists of germline mutations with lists of known SNPs, to running complex computational methods to identify protein-protein interaction sub-networks affected by mutations. Some cancer analysis workflows opt to develop these functionalities in-house, while others delegate them to third party software with the implicit burdens of installation and configuration. Table 1 lists some software resources that are useful when implementing analysis workflows, and succinctly describes their functionality and availability.

The functionalities required in a genome analysis workflow can be divided into four classes, depending on how they are accessed (Table 3): via web services, local or browser based applications, command line tools, or application programming interfaces (APIs). It is not uncommon for resources to make their data and functionalities available in several ways, a trend that is already evident in databases like Ensembl, where the information can be examined using the web interface, downloaded via the BioMart web service, batch downloaded from an FTP server, or queried through the PERL API.

**Table 3:** Types of third party software and their general characteristics.

Software type	Installation	User friendly	Scriptable	Reusable^1^
Browser app.	NO	YES	NO^2^	NO
Web server	NO	NO	YES	NO
Local app	YES	YES	NO^3^	NO
Command line	YES	NO	YES	YES^4^
API	YES	NO	YES	YES

1 Reusable means that the code, in whole or in part, can be reused for some other purpose.

2 May be scriptable using web scraping.

3 May support some macro definitions and batch processing.

4 If the source code is provided and is easy to pick apart.

Bioinformaticians should strive to make their resources widely available to allow others to use them in the most convenient manner. In function of the workflow's characteristics, some accessibility modes (*e.g.*, web service, local application, or API) will be more convenient than others. For example, if a relatively systematic workflow has to be applied to a batch of datasets, then command-line tools are very convenient as they are easy to script. Because a cancer genome analysis pipeline may require several connected analytical steps, it is important to be able to script them to avoid manual operations, thereby guaranteeing the sustainability and reproducibility of the results. Conversely, if the user is concerned with the analysis of just one dataset but interpretation of the results requires more careful examination, visual interfaces such as browser-based applications may be the most convenient end-user interface, as these can link the results to knowledge databases to set the context.

## 5. Workflow Enactment Tools and Visual Interfaces

Given the complexity of cancer genome analysis, it is worth discussing how to design and execute (enact) workflows, which may become very elaborate. Workflows can be thought of as analysis recipes, whereby each analysis entails enacting that workflow using new data. Ideally a workflow should be comprehensive and cover the complete analysis process from the raw data to the final results. These workflows may involve processing different types of data and may require specific adaptations for the analysis of certain types of experiments. Often, parts of the analysis will be repeated in a different context and thus, one of the objectives of workflow enactment tools is to reuse code efficiently. A number of systems have been designed to facilitate the construction of workflows (*e.g.*, Taverna [21] and Galaxy [22], which both offer visual interfaces to orchestrate workflows across a very wide range of available functionalities).

Although visual workflow enactment approaches have become reasonably popular, they still have several important limitations. Firstly, despite recent efforts, these approaches remain overly complex for non-bioinformaticians. Secondly, they are quite inflexible in terms of the presentation and exploration of the results, and thus, understanding the results requires the user to do additional work outside of the system. Finally, the expressiveness of these approaches is limited when compared with general purpose programming languages. Experienced developers will find them of limited utility, and prefer to have their functionalities accessible by APIs derived from general purpose programming languages.

The information presented to the user needs to closely match his/her needs, especially in more translational settings. Too much information may mask important conclusions, while too little may leave the user unsure as to the validity of their findings. This further emphasizes the need to customize workflows and the manner in which results are displayed, in order to best fit these aspects to the particularities of each user.

In a more academic setting, close collaboration between the researcher and the bioinformatician facilitates the development of custom interfaces that can better adapt to given datasets, and answer the very specific questions that may arise during data exploration. In our institution, we use a programmatic workflow enactment system that orchestrates a wide variety of tasks, ranging from coordinate mapping to enrichment analysis. This system is controlled via a browser application designed to rapidly produce custom reports using a template-based HTML report generation system. It is a system that was developed entirely in-house but that makes use of third party software, allowing us to address the requirements of our collaborators in a timely manner.

## 6. Summary

Cancer genome analysis involves the manipulation of large datasets and the application of complex methods. The heterogeneity of the data and the disparity of the software implementations represent an additional layer of complexity, which requires the use of systems that can be easily adapted and reconfigured. Additionally, interpretation of the results in terms of specific biological questions is more effective if done in close collaboration with experts in the field. This represents a specific challenge for software development in terms of interactivity and representation standards. Cancer genome analysis systems need to be capable of conveniently managing this complexity and of adapting to the specific characteristics of each analysis.

Finally, it is worth noting that bioinformatics systems will soon have to move beyond the current research environments and into clinical settings, a challenge that will involve more industrial development that can better cope with issues of sustainability, robustness and accreditation, while still incorporating the latest bioinformatics components that will continue to be generated in research laboratories. This constitutes a new and exciting frontier for bioinformatics software developers.

## 7. Exercise Questions

- Name three general issues that bioinformaticians face when analyzing cancer genome data?
- What are the four main tasks in cancer genome analysis in a clinical setting once the primary analysis has been performed?
- Why is it important to use the correct genome build?
- What do we mean by driver mutation?
- There are two key principles that help determine driver mutations *in-silico*. What are they?
- Give several reasons why point mutations in coding regions are so important.
- Name three issues that challenge the assumptions made by the standard pathway enrichment analysis tools when applied to genomic mutations.
- Discuss the problems that arise with identifiers when integrating information across different databases.
- Why are command line tools generally more convenient than browser-based applications for processing a batch analyses?
- How would an application aimed at researchers differ from one aimed at clinicians in terms of the information presented?

Answers to the Exercises can be found in Text S1.

**Further Reading**

- Weinberg RA (2006) The biology of cancer. 1st ed. Garland Science. 850 p.
- Ng PC, Murray SS, Levy S, Venter JC (2009) An agenda for personalized medicine. Nature 461: 724–726. doi:10.1038/461724a.
- Fernald GH, Capriotti E, Daneshjou R, Karczewski KJ, Altman RB (2011) Bioinformatics challenges for personalized medicine. Bioinformatics 27: 1741–1748. doi:10.1093/bioinformatics/btr295.
- Valencia A, Hidalgo M (2012) Getting personalized cancer genome analysis into the clinic: the challenges in bioinformatics. Genome Med 4: 61. doi:10.1186/gm362.
- Whirl-Carrillo M, McDonagh EM, Hebert JM, Gong L, Sangkuhl K, et al. (2012) Pharmacogenomics knowledge for personalized medicine. Clin Pharmacol Ther 92: 414–417. doi:10.1038/clpt.2012.96.
- Baudot A, de la Torre V, Valencia A (2010) Mutated genes, pathways and processes in tumours. EMBO Rep 11: 805–810. doi:10.1038/embor.2010.133.
- Huang DW, Sherman BT, Lempicki RA (2009) Bioinformatics enrichment tools: paths toward the comprehensive functional analysis of large gene lists. Nucl Acids Res 37: 1–13. doi:10.1093/nar/gkn923.
- Khatri P, Sirota M, Butte AJ (2012) Ten years of pathway analysis: current approaches and outstanding challenges. PLoS Comput Biol 8: e1002375. doi:10.1371/journal.pcbi.1002375.
- Stein L (2002) Creating a bioinformatics nation. Nature 417: 119–120. doi:10.1038/417119a.

## Supporting Information

Text S1 Answers to Exercises
(DOCX)

## References

[1] Hudson TJ, Anderson W, Artez A, Barker AD, Bell C, et al (2010) International network of cancer genome projects. Nature 464: 993–998 doi:10.1038/nature08987. 20393554 10.1038/nature08987 PMC2902243

[2] Roychowdhury S, Iyer MK, Robinson DR, Lonigro RJ, Wu Y-M, et al (2011) Personalized oncology through integrative high-throughput sequencing: a pilot study. Sci Transl Med 3: 111ra121 doi:10.1126/scitranslmed.3003161. 10.1126/scitranslmed.3003161 PMC3476478 22133722

[3] Villarroel MC, Rajeshkumar NV, Garrido-Laguna I, De Jesus-Acosta A, Jones S, et al (2011) Personalizing cancer treatment in the age of global genomic analyses: PALB2 gene mutations and the response to DNA damaging agents in pancreatic cancer. Mol Cancer Ther 10: 3–8 doi:10.1158/1535-7163.MCT-10-0893. 21135251 10.1158/1535-7163.MCT-10-0893 PMC3307340

[4] Valencia A, Hidalgo M (2012) Getting personalized cancer genome analysis into the clinic: the challenges in bioinformatics. Genome Med 4: 61 doi:10.1186/gm362. 22839973 10.1186/gm362 PMC3580417

[5] Baudot A, Real FX, Izarzugaza JMG, Valencia A (2009) From cancer genomes to cancer models: bridging the gaps. EMBO Rep 10: 359–366 doi:10.1038/embor.2009.46. 19305388 10.1038/embor.2009.46 PMC2672900

[6] Andrewes C (1964) Tumour-viruses and Virus-tumours. Br Med J 1: 653–658. 14096457 10.1136/bmj.1.5384.653 PMC1813804

[7] Nielsen R, Paul JS, Albrechtsen A, Song YS (2011) Genotype and SNP calling from next-generation sequencing data. Nature Reviews Genetics 12: 443–451 doi:10.1038/nrg2986. 10.1038/nrg2986 PMC3593722 21587300

[8] Metzker ML (2010) Sequencing technologies - the next generation. Nat Rev Genet 11: 31–46 doi:10.1038/nrg2626. 19997069 10.1038/nrg2626

[9] Quesada V, Conde L, Villamor N, Ordóñez GR, Jares P, et al (2011) Exome sequencing identifies recurrent mutations of the splicing factor SF3B1 gene in chronic lymphocytic leukemia. Nat Genet 44: 47–52 doi:10.1038/ng.1032. 22158541 10.1038/ng.1032

[10] Kulis M, Heath S, Bibikova M, Queirós AC, Navarro A, et al (2012) Epigenomic analysis detects widespread gene-body DNA hypomethylation in chronic lymphocytic leukemia. Nat Genet 44): 1236–1242 doi:10.1038/ng.2443. 23064414 10.1038/ng.2443

[11] Chuang H-Y, Rassenti L, Salcedo M, Licon K, Kohlmann A, et al (2012) Subnetwork-based analysis of chronic lymphocytic leukemia identifies pathways that associate with disease progression. Blood 120: 2639–2649 doi:10.1182/blood-2012-03-416461. 22837534 10.1182/blood-2012-03-416461 PMC3460686

[12] The Cancer Genome Atlas Network (2012) Comprehensive molecular portraits of human breast tumours. Nature 490: 61–70 doi:10.1038/nature11412. 23000897 10.1038/nature11412 PMC3465532

[13] Chen R, Mias GI, Li-Pook-Than J, Jiang L, Lam HYK, et al (2012) Personal omics profiling reveals dynamic molecular and medical phenotypes. Cell 148: 1293–1307 doi:10.1016/j.cell.2012.02.009. 22424236 10.1016/j.cell.2012.02.009 PMC3341616

[14] Fröhling S, Scholl C, Levine RL, Loriaux M, Boggon TJ, et al (2007) Identification of driver and passenger mutations of FLT3 by high-throughput DNA sequence analysis and functional assessment of candidate alleles. Cancer Cell 12: 501–513 doi:10.1016/j.ccr.2007.11.005. 18068628 10.1016/j.ccr.2007.11.005

[15] Boris Reva YACS (2011) Predicting the functional impact of protein mutations: application to cancer genomics. Nucleic Acids Res 39: e118 doi:10.1093/nar/gkr407. 21727090 10.1093/nar/gkr407 PMC3177186

[16] Bernstein BE, Birney E, Dunham I, Green ED, Gunter C, et al (2012) An integrated encyclopedia of DNA elements in the human genome. Nature 489: 57–74 doi:10.1038/nature11247. 22955616 10.1038/nature11247 PMC3439153

[17] Wang L, Lawrence MS, Wan Y, Stojanov P, Sougnez C, et al (2011) SF3B1 and other novel cancer genes in chronic lymphocytic leukemia. N Engl J Med 365: 2497–2506 doi:10.1056/NEJMoa1109016. 22150006 10.1056/NEJMoa1109016 PMC3685413

[18] Damm F, Nguyen-Khac F, Fontenay M, Bernard OA (2012) Spliceosome and other novel mutations in chronic lymphocytic leukemia, and myeloid malignancies. Leukemia 26: 2027–2031. 22484420 10.1038/leu.2012.86

[19] Subramanian A, Tamayo P, Mootha VK, Mukherjee S, Ebert BL, et al (2005) Gene set enrichment analysis: a knowledge-based approach for interpreting genome-wide expression profiles. Proc Natl Acad Sci USA 102: 15545–15550 doi:10.1073/pnas.0506580102. 16199517 10.1073/pnas.0506580102 PMC1239896

[20] Glaab E, Baudot A, Krasnogor N, Schneider R, Valencia A (2012) EnrichNet: network-based gene set enrichment analysis. Bioinformatics 28: i451–i457 doi:10.1093/bioinformatics/bts389. 22962466 10.1093/bioinformatics/bts389 PMC3436816

[21] Hull D, Wolstencroft K, Stevens R, Goble C, Pocock MR, et al (2006) Taverna: a tool for building and running workflows of services. Nucleic Acids Res 34: W729–W732 doi:10.1093/nar/gkl320. 16845108 10.1093/nar/gkl320 PMC1538887

[22] Giardine B, Riemer C, Hardison RC, Burhans R, Elnitski L, et al (2005) Galaxy: a platform for interactive large-scale genome analysis. Genome Res 15: 1451–1455 doi:10.1101/gr.4086505. 16169926 10.1101/gr.4086505 PMC1240089

