# Supplementary material for: Chapter 14: Cancer Genome Analysis
Source: PLoS Comput Biol. 2012 Dec 27;8(12):e1002824. doi: 10.1371/journal.pcbi.1002824 (PMC3531315; doi:10.1371/journal.pcbi.1002824)
Supplement: Text S1 — Answers to Exercises (DOCX) [file pcbi.1002824.s001.docx]

Exercise Questions and Answers

## I. Name three general issues that bioinformaticians face when analyzing cancer genome data?

1. The heterogeneity among the types of data that need to be examined.
2. The wide range of software and database resources used.
3. The expertise required in multiple areas of biology to interpret the results.

## II. What are the four main tasks in cancer genome analysis in a clinical setting once the primary analysis has been performed?

1. Coordinate mapping.
2. Pathogenicity predictions and selection of driver mutations.
3. Functional interpretation.
4. Presentation of actionable results.

## III. Why is it important to use the correct genome build?

Coordinate mapping is the first step in the interpretation of somatic mutations. If the wrong genome build is used, the locations of genes will almost certainly be shifted, resulting in incorrect mapping.

## IV. What do we mean by driver mutation?

A driver mutation is a mutation that is involved in the onset or progression of tumors.

## V. There are two key principles that help determine driver mutations *in-silico*. What are they?

1. Tumors exhibit traces of mutation selection that can be detected by statistical analysis.
2. Driver mutations must cause some functional alteration, which can be assessed by pathogenicity predictions.

## VI. Give several reasons why point mutations in coding regions are so important.

We have a better understanding of their potential pathogenicity than that of other types of mutations.

Exome sequencing is still cheaper than whole-genome sequencing.

The therapeutic implications of these mutations are clearer.

## VII. Name three issues that challenge the assumptions made by the standard pathway enrichment analysis tools when applied to genomic mutations.

1. They do not account for the number of mutations per gene.
2. They do not account for variation between genes in the probability of undergoing mutation.
3. They do not account for mutations affecting several overlapping genes

## VIII. Discuss the problems that arise with identifiers when integrating information across different databases.

Different resources use different identifiers to refer to their entities. The need to map between different identifier formats may introduce technical incompatibilities between resources. In addition, the differences between the equivalent entities in different resources may go beyond the identifiers used, and they may reflect slightly different definitions of these entities.

## IX. Why are command line tools generally more convenient than browser-based applications for processing a batch analyses?

Command line tools are easier to script, thereby avoiding the tedious manual operations that could be a potential source of error.

## X. How would an application aimed at researchers differ from one aimed at clinicians in terms of the information presented?

An application aimed at researchers would include more contextual information and would support more exploratory use. An application aimed at clinicians would focus more on the actionable aspects and include only the information required to aid decision-making without adding unnecessary clutter.
